# Initial experience with AcQMap catheter for treatment of persistent atrial fibrillation and atypical atrial flutter

**DOI:** 10.1007/s12471-021-01636-w

**Published:** 2021-10-26

**Authors:** M. Liebregts, M. C. E. F. Wijffels, M. N. Klaver, V. F. van Dijk, J. C. Balt, L. V. A. Boersma

**Affiliations:** 1 Department of Cardiology, St. Antonius Hospital, Nieuwegein, The Netherlands; 2 Department of Cardiology, Amsterdam University Medical Centers, Amsterdam, The Netherlands

**Keywords:** Atrial fibrillation, Atrial flutter, Mapping system, Catheter ablation

## Abstract

**Introduction:**

The AcQMap High Resolution Imaging and Mapping System was recently introduced. This system provides 3D maps of electrical activation across an ultrasound-acquired atrial surface.

**Methods:**

We evaluated the feasibility and the acute and short-term efficacy and safety of this novel system for ablation of persistent atrial fibrillation (AF) and atypical atrial flutter.

**Results:**

A total of 21 consecutive patients (age (mean ± standard deviation) 62 ± 8 years, 23% female) underwent catheter ablation with the use of the AcQMap System. Fourteen patients (67%) were treated for persistent AF and 7 patients (33%) for atypical atrial flutter. Eighteen patients (86%) had undergone at least one prior ablation procedure. Acute success, defined as sinus rhythm without the ability to provoke the clinical arrhythmia, was achieved in 17 patients (81%). At 12 months, 4 patients treated for persistent AF (29%) and 4 patients treated for atypical flutter (57%) remained in sinus rhythm. Complications included hemiparesis, for which intra-arterial thrombolysis was given with subsequent good clinical outcome (*n* = 1), and complete atrioventricular block, for which a permanent pacemaker was implanted (*n* = 2). No major complications attributable to the mapping system occurred.

**Conclusion:**

The AcQMap System is able to provide fast, high-resolution activation maps of persistent AF and atypical atrial flutter. Despite a high acute success rate, the recurrence rate of persistent AF was relatively high. This may be due to the selection of the patients with therapy-resistant arrhythmias and limited experience in the optimal use of this mapping system that is still under development.

**Supplementary Information:**

The online version of this article (10.1007/s12471-021-01636-w) contains supplementary material, which is available to authorized users.

## What’s new?

- This is the first study describing real-world outcomes in patients treated for persistent atrial fibrillation (AF) and atypical atrial flutter with the use of the AcQMap system.
- The AcQMap system provided a high acute success rate (81%).
- No recurrence of clinical arrhythmia was observed in 86% of the patients treated for atypical atrial flutter during a follow-up of 12 months.
- Only 29% of the patients treated for persistent AF remained in sinus rhythm after a follow-up of 12 months.

## Introduction

Pulmonary vein isolation (PVI) for the treatment of paroxysmal atrial fibrillation (AF) and cavotricuspid isthmus ablation for the treatment of typical atrial flutter are straightforward procedures with high success rates [1, 2]. Additional non-PV triggers responsible for driving and maintaining AF are thought to exist in persistent AF [3–5]. This explains why PVI alone renders a much lower success rate in these patients [6, 7]. Current point-by-point mapping systems are unable to accurately display complex arrhythmias because of the sequential nature by which such systems acquire, post-process and display voltage-based contact signals. Therefore, they are not well suited to locate and eliminate the drivers and maintenance mechanisms of persistent AF.

We report our first experience with a novel non-contact ultrasound array catheter for mapping and treating persistent AF and atypical atrial flutter.

## Methods

### Patient selection and study design

All patients who were treated for persistent AF or atypical atrial flutter with the use of the AcQMap High Resolution Imaging and Mapping System (Acutus Medical, Carlsbad, CA, USA) at the St. Antonius Hospital Nieuwegein, the Netherlands between December 2016 and December 2018 were included. The procedures were conducted by one or two operators (MCEFW and/or LVAB). All patients had their first clinical check-up at 3 months and were followed up for a maximum of 12 months. Patients who underwent a redo procedure were excluded from further follow-up. Baseline characteristics, procedural data and outcomes were retrospectively collected from hospital patient records.

### Description of the system

The AcQMap System has the ability to rapidly generate 3D ultrasound maps of heart chambers overlaid with high-resolution activation maps. The electrical activation can be shown as voltage or dipole density. Voltage maps are made up of the summation of local charges arising from cardiac cells all over the myocardium. Dipole density maps (Coulombs/cm^2^) represent the magnitude of these sources upon the endocardial surface and are therefore more precise [8]. The AcQMap System acquires dipole density from non-contact sensing of cardiac voltage within the heart chamber as a whole. This results in a global view of the conduction pattern of every activation cycle and permits the acquisition of consecutive activation maps on a beat-to-beat basis [9].

**Fig. 1:**
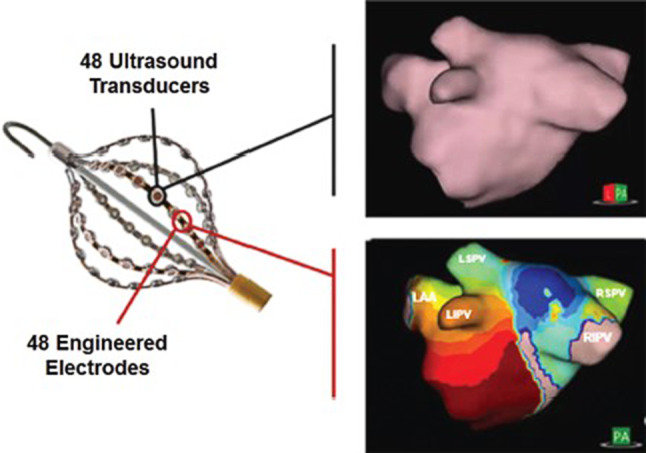
AcQMap catheter. The six splines of the catheter have 48 ultrasound transducers for anatomy reconstruction (upper right panel) and 48 electrodes for recording biopotential signals used to create propagation history maps (lower right panel). This figure was reprinted with permission from [10]

The diagnostic recording catheter (AcQMap 3D Imaging and Mapping Catheter) consists of six splines, which are populated with eight ultrasound transducers and eight biopotential electrodes each, making up a total of 48 sensors of each type. The catheter is deployed by the user into the left or right atrium as a spheroidal basket (Fig. 1). Patch electrodes are attached to the patient to provide localisation data to the system. To create an anatomical map, the user continuously rotates the catheter back and forth for approximately 3 min. During this time, the ultrasound subsystem samples the endocardial surface at a rate of up to 115,000 surface points per minute. The location of each surface point is determined by the time it takes an acoustic wave to travel from an ultrasound transducer to the endocardial surface and back. The ultrasound point-set is subsequently transformed into a surface mesh of the atrial chamber. After minimal post processing (removal of unnecessary points, labelling of anatomical structures), the map is ready to be used as a canvas for the second part.

For this part, the basket is placed in the centre of the atrium and a quadripolar catheter is placed in the coronary sinus as electrical reference. Then, unipolar intracardiac potentials across the endocardial surface are measured by the 48 biopotential electrodes at a rate of 150,000 samples per second. This again takes approximately 2–3 min, depending on the cycle length chosen. After data are recorded, inverse and forward algorithms are applied. For example, the QRS complex is removed to enable continuous display of activation wave fronts. The calculated data are spatially and temporally applied to the final processed surface anatomy as either a dipole density or voltage depolarisation map, or a propagation-history map. In a depolarisation map, the red region depicts the negative phase of dipole density, which, when animated, is associated with propagation of depolarisation. In the propagation-history map, red represents the leading edge of the activation wave front, with the trailing colour bands showing past locations of the wave front (Fig. 1, [9]).

## Results

A total of 21 patients (age (mean ± standard deviation [SD]) 62 ± 8 years, 23% female) were included. Fourteen patients (67%) were treated for persistent AF and 7 (33%) for atypical atrial flutter. Ablations were conducted with an irrigated ablation catheter with contact force measurement.

### Patients treated for persistent atrial fibrillation

#### Clinical characteristics

Baseline characteristics of the 14 patients treated for persistent AF (mean ± SD age 64 ± 8 years, 21% female) are shown in Tab. 1. Eleven patients (79%) had undergone at least one prior ablation procedure and 4 (29%) had undergone at least two prior ablation procedures. Mean ± SD left atrial volume index was 36 ± 8 mL/m^2^.

**Table 1:** Baseline characteristics of 14 patients treated for persistent atrial fibrillation and 7 patients treated for atypical atrial flutter

Patient number	Diagnosis	Age, years	Sex	Clinical history	LAVI, mL/m^2^
1	AF	59	M	PVI, re-PVI, VCS isolation	NA
2	AF	68	F	PVI	33
3	AFL	69	M	Mini-Maze (PVI + box + trigonum line + bicaval line)	45
4	AF	47	M	PVI	49
5	AF	70	F	PVI	32
6	AF	54	M	None	25
7	AF	52	M	None	43
8	AF	61	M	None	27
9	AFL	68	M	PVI	NA
10	AF	57	M	PVI, mini-Maze (PVI + box + ganglion plexus ablation)	44
11	AF	77	M	PVI	NA
12	AF	66	F	PVI, mini-Maze (PVI + box + trigonum line + bicaval line + RA line)	33
13	AFL	69	M	PVI, re-PVI, mini-Maze (PVI + box + trigonum line)	67
14	AFL	68	M	PVI, CTI ablation, mini-Maze	NA
15	AF	65	M	Mini-Maze (PVI + box + trigonum line + bicaval line + RA line)	43
16	AFL	78	F	PVI, re-PVI, CTI ablation, mini-Maze	20
17	AF	68	M	PVI	29
18	AF	75	M	PVI	NA
19	AFL	61	F	PVI, mini-Maze (PVI + box + trigonum line + bicaval line)	30
20	AFL	66	M	PVI, re-PVI, CTI ablation, re-re-PVI, mini-Maze (PVI + box)	32
21	AF	71	M	PVI, re-PVI, CryoMaze (PVI + box + trigonum line)	46

*AF* atrial fibrillation, *AFL* atrial flutter, *PVI* pulmonary vein isolation, *VCS* vena cava superior,* LAVI* left atrial volume index, *RA* right atrial, *CTI* cavotricuspid isthmus

#### The procedure

**Fig. 2:**
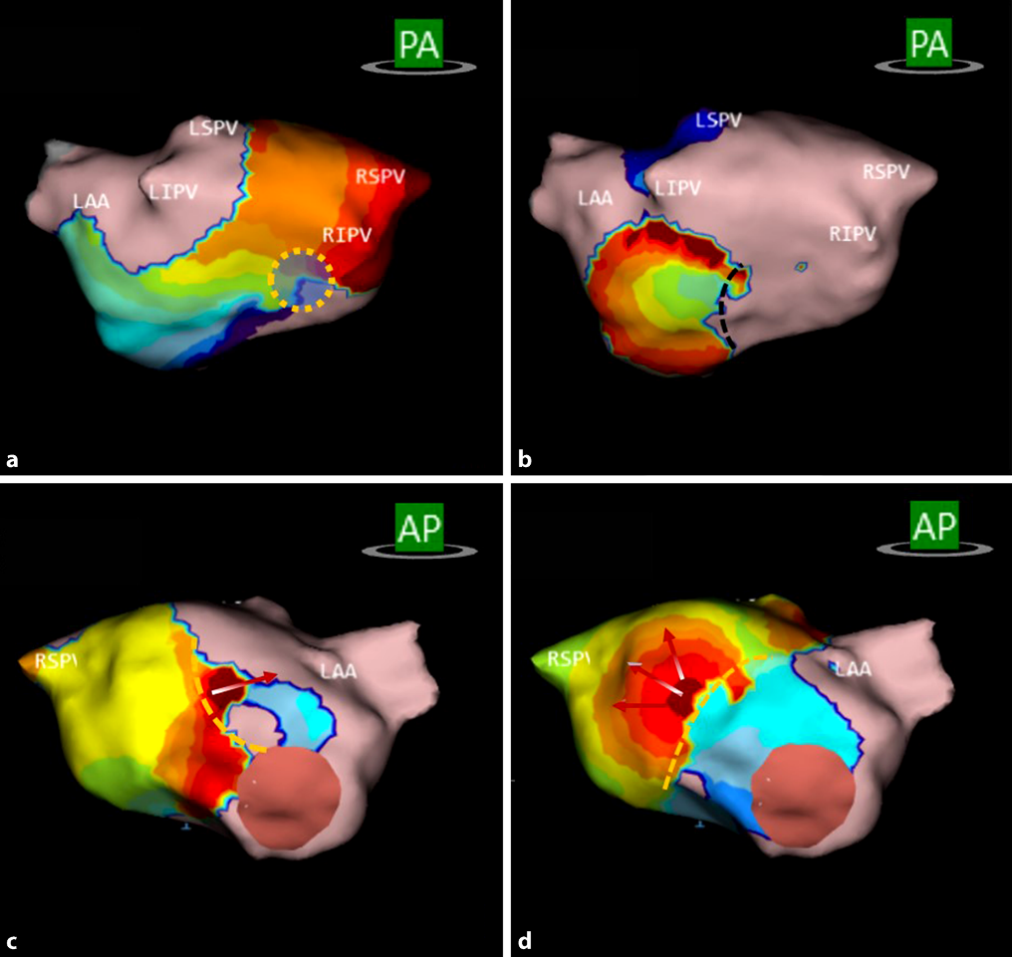
Three activation patterns of interest in patient 21. All panels show propagation-history maps of the left atrium, which use bands of colour to show location and velocity of leading edge of wave front over a set period of time. Red colour indicates leading edge of wave front, with trailing colour bands showing earlier locations. Width of colour bands indicates conduction velocity of wave fronts; wider bands are indicative of fast conduction and narrow bands of slow conduction. **a** Localised rotational activation (spiralling around a small, confined zone, indicated by dotted *yellow circle*) in the posterior wall of left atrium (*LA*). **b** Focal activation in posterior wall of LA. **c**, **d** Localised irregular activation (entry/exit through confined zone, indicated by *red arrows*) in anterior wall of LA. *PA* posteroanterior, *AP* anteroposterior, *LAA* left atrial appendage, *LIPV* left inferior pulmonary vein, *LSPV* left superior pulmonary vein, *RIPV* right inferior pulmonary vein, *RSPV* right superior pulmonary vein

Median (interquartile range [IQR]) procedural time was 180 min (156–218), and median (IQR) radiofrequency ablation time was 44 min (21–79) (see Tab. S1 in the Electronic Supplementary Material). All patients had AF at the start of the procedure. Following PVI or re-PVI, atrial activation patterns of interest (APIs) were targeted. These included focal, localised rotational activation (spiralling around a small, confined zone ≥ 270°) and localised irregular activation (entry/exit through and pivoting around a confined zone). These APIs were ablated separately at each remapped stage and, where possible, connected to near PVI circumferential lesions and/or anatomical barriers. Fig. 2 shows examples of the three mechanisms in patient 21. The number of mechanisms in all 14 patients by location is shown in Fig. S1 (see Electronic Supplementary Material). If conversion to sinus rhythm did not occur after ablation of all APIs, electrocardioversion was performed.

#### Procedural success

Two patients (patients 10 and 12) converted to sinus rhythm during ablation. At the end of the procedure, a total of 11 patients (79%) were in sinus rhythm (see Tab. S1 in the Electronic Supplementary Material). In the remaining 3 patients, AF persisted despite electrocardioversion. In one of these patients, the right inferior pulmonary vein had a very low take-off, which made it impossible to isolate. In the other 2 patients APIs kept on returning despite extensive ablation.

At the 3‑month follow-up point, 5 patients (36%) were in sinus rhythm (Tab. 2). Of the patients with no prior ablation procedure or only one prior PVI, 3 (33%) were in sinus rhythm after 3 months. Of the patients with multiple prior ablation procedures, 2 (40%) were in sinus rhythm after 3 months. Five patients (36%) underwent a redo procedure around 6 months of follow-up. At the 12-month follow-up point, 4 patients (without redo procedure) (29%) remained in sinus rhythm.

**Table 2:** Outcomes of 14 patients treated for persistent atrial fibrillation

Patient number	3 months	6 months	9 months	12 months
1	AF (ECG)	AF (ECG)	AF (ECG)	AF (ECG)
2	SR (ECG)^a^	NA	SR (Holter)	SR (ECG)
4	AFL (ECG)	Redo ablation	–	–
5	AF (ECG)	Mini-Maze	–	–
6	SR (ECG)^a^	SR (Holter)^a^	SR (ECG)	SR (ECG)
7	AF (ECG)	SR (ECG)^a^	Paroxysmal AF (Holter)	SR (Holter)^a^
8	AFL (ECG)	Redo ablation	–	–
10	SR (pacemaker)^a^	NA	SR (pacemaker)	SR (pacemaker)
11	SR (ECG)^a^	SR (ECG)	SR (ECG)	AF (ECG)
12	SR (ECG)	NA	NA	SR (ECG)
15	AF (ECG)	Redo ablation	–	–
17	AF (ECG)	Persistent AF (pacemaker)	Persistent AF (pacemaker)	Persistent AF (pacemaker)
18	AF (ECG)	Mini-Maze	–	–
21	AF (ECG)	AFL (ECG)	NA	NA

*AF* atrial fibrillation, *ECG* electrocardiography, *SR* sinus rhythm,* NA* not available, *AFL* atrial flutter

^a^ Patient was on antiarrhythmic drugs

### Patients treated for atypical flutter

#### Clinical characteristics

Baseline characteristics of the 7 patients treated for atypical flutter (mean ± SD age 68 ± 5 years, 29% female) are shown in Tab. 1. All patients had undergone at least one prior ablation procedure and 5 (71%) had undergone at least two procedures. Mean ± SD left atrial volume index was 38 ± 18 mL/m^2^.

#### The procedure

**Fig. 3:**
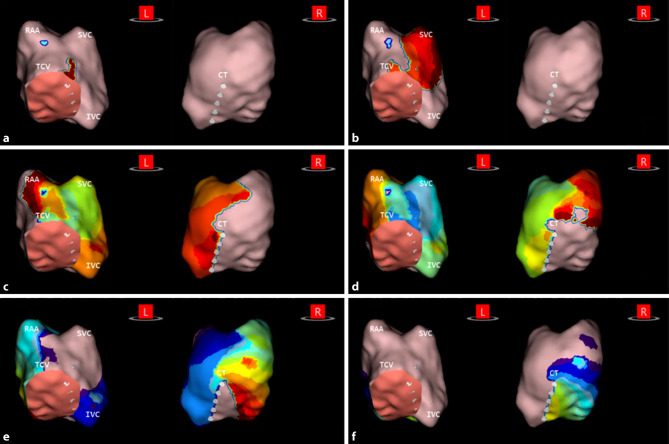
Example of right-sided atypical flutter in patient 16. Propagation-history map of right atrium, which uses bands of colour to show location and velocity of leading edge of wave front over a set period of time. Red colour indicates leading edge of wave front, with trailing colour bands showing earlier locations. Width of colour bands indicates conduction velocity of wave fronts; wider bands are indicative of fast conduction and narrow bands of slow conduction. **a** Focal activation at tricuspid annulus. **b** Propagation of wavefront towards superior vena cava (*SVC*). **c** Separation into two wavefronts towards inferior septum and lateral wall, respectively. **d** Propagation of two wavefronts down septum and lateral wall. **e**, **f** Collision of two wavefronts at cavotricuspid isthmus. *L* left, *R* right, *RAA* right atrial appendage, *TCV* tricuspid valve,* IVC* inferior vena cava, *CT* crista terminalis

Median (IQR) procedural time was 205 min (190–215), and median (IQR) radiofrequency ablation time was 11 min (10–16) (see Tab. S1 in the Electronic Supplementary Material). Five patients had atrial flutter at the start of the procedure. In the remaining 2 patients, atrial flutter was provoked by incremental atrial pacing. Median (IQR) cycle length was 251 ms (200–260). In 3 patients, ablation was performed in the left atrium, in 3 patients in the right atrium, and in 1 patient in both. Fig. 3 shows an example of a right-sided atypical flutter in patient 16 (for the complete video, see Electronic Supplementary Material).

#### Procedural success

Acute success, defined as sinus rhythm without the ability to provoke the clinical arrhythmia, was achieved in 6 patients (86%) (see Tab. S1 in the Electronic Supplementary Material). At the 3‑month follow-up point, 4 patients (57%) were in sinus rhythm, and these patients remained in sinus rhythm during 12 months of follow-up (Tab. 3). One patient (14%) had a recurrence of the clinical arrhythmia and later developed AF. The remaining 2 patients also developed AF, for which one of them underwent a redo ablation.

**Table 3:** Outcomes of 7 patients treated for atypical atrial flutter

Patient number	3 months	6 months	9 months	12 months
3	SR (ECG)	NA	SR (ECG)	SR
9	SR (ECG)^a^	SR (ECG)	SR (Holter)	SR (ECG)
13	SR (ICD)	SR (ICD)	SR (ICD)	SR (ICD)
14	AFL (ECG)	NA	NA	AF (ECG)
16	Persistent AF (pacemaker)	NA	NA	Persistent AF (pacemaker)
19	Redo ablation	–	–	–
20	SR (pacemaker)	SR (pacemaker)	SR (pacemaker)	SR (pacemaker)

*SR* sinus rhythm, *NA* not available, *ECG* electrocardiography, *ICD* internal cardiac defibrillator, *AFL* atrial flutter, *AF* atrial fibrillation

^a^ Patient was on antiarrhythmic drugs

### Complications

All complications are listed in Table S1 (see Electronic Supplementary Material). There were 2 patients (10%) with major complications attributable to radiofrequency ablation. Both were treated for an atypical flutter; one patient developed hemiparesis for which intra-arterial thrombolysis was given with subsequent good clinical outcome. The same patient and one other patient developed complete atrioventricular block, for which a permanent pacemaker was implanted. No major complications attributable to the mapping system occurred.

## Discussion

Persistent AF is a complex arrhythmia, with drivers and maintenance mechanisms outside the pulmonary veins [3–5]. Conventional point-by-point mapping is inadequate to understand these mechanisms, and empiric ablation has been disappointing [6]. Global mapping of AF could overcome the limitations of point-by-point mapping and facilitate a patient-tailored approach.

The first noncontact global mapping system was the EnSite Array (Abbott/St. Jude Medical Inc., St. Paul, MN, USA), which comprises 64 unipolar electrodes [11]. It displays calculated endocardial unipolar electrograms on a surface that is physically traced by a conventional catheter. The main limitation of this technology is that the precision of the calculated electrograms depends on the distance from the endocardial surface. Moreover, it has only been validated for distances < 40 mm [12].

The FIRMap Catheter (with the use of the Topera Rhythm View 3D Mapping System, Abbott/Topera Medical, Palo Alto, CA, USA) was the first to identify spiral rotors and focal drivers of AF and facilitate patient-specific ablation in humans. The CONFIRM study showed an acute success rate of 86% and freedom from AF in 83% of the patients after a median follow-up period of 273 days [13]. However, more recent studies have failed to achieve acute and long-term outcomes similar to those seen in the CONFIRM study [14–18]. A possible explanation for the disappointing results from these studies is that the FIRMap Catheter is a contact basket, which is meant to be a ‘one size fits all’. Several studies have reported poor contact by the basket catheter electrodes and clustering of splines [15, 18].

The AcQMap High Resolution Imaging and Mapping System is the latest addition to the arsenal of global AF mapping systems. This system provides 3D maps of electrical activation across an ultrasound-acquired atrial surface. The acquisition of the ultrasound surface map takes 2–3 min, after which consecutive activation maps can be generated on a beat-to-beat basis. Another novel aspect of the system is that it can generate dipole density maps in addition to the conventional voltage maps.

A dipole consists of two oppositely charged particles separated by a very small distance. Each time a cell is stimulated, ions move across the cellular membrane, causing a small dipolar imbalance in the adjoining extracellular medium. The combined and consecutive activation of multiple cells forms a macroscopic double layer of dipoles that directly represents the wave front and, in turn, generates the cardiac potential field, measured in volts. The differences between voltage and dipole density lie in both the averaging effect of ‘spatial summation’ and in the volume of space occupied by each. Spatial averaging causes the distribution of voltage to extend far beyond the compact, physical boundary of the charges. It also smooths out some of the localised details of the geometric shape of the wave front. This explains why it is possible to measure the heart’s electrocardiographic signals with an ECG, although with significantly less spatial detail than that of intracardiac electrograms [19].

The AcQMap System is able to generate dipole density maps because it can simultaneously measure the endocardial potential field and the anatomic surface. This enables spatially localised and temporally animated derivation of the dipolar charge sources on the endocardial surface, which can be displayed as waves of activation across a reconstructed 3D surface through time.

### Recurrence rate

In our experience, the AcQMap System is user-friendly and safe. Atypical flutter circuits and APIs were easily identified, and the acute success rate was high. Of the patients treated for atypical flutter, only one patient had a recurrence of the clinical arrhythmia during follow-up. The outcome of the patients treated for persistent AF was less optimal. After 12 months, only 29% of the patients were in sinus rhythm. One possible explanation for this high recurrence rate is that the studied population had already been found to be therapy-resistant (79% had undergone at least one prior ablation procedure). Moreover, the recently published UNCOVER trial, which included 127 patients with persistent AF who underwent *de novo* catheter ablation using the AcQMap System, showed a single-procedure freedom from AF rate of 73% at 12 months [20].

Another possible, but less hopeful, explanation for the high recurrence rate in our cohort is that persistent AF is not the resultant of single or small numbers of stable localised APIs, but that their location varies over time as the substrate may keep deteriorating. Observations by Haissaguerre et al. point in this direction [21, 22]. They used a body surface mapping system with 252 external electrodes in combination with computed tomography to record biatrial unipolar electrograms and create simultaneous biatrial 3D activation maps. These maps showed incessantly changing beat-to-beat wavefronts and varying spatiotemporal behaviour of driver activities. The rotors they found had a median duration of 2.6 rotations, as opposed to the minutes to hours in the CONFIRM study. The authors also found that the number of targeted driver regions increased with the duration of persistent AF (2 in patients presenting in sinus rhythm, 3 in AF lasting 1 to 3 months, 4 in AF lasting 4 to 6 months, and 6 in AF lasting longer), while the termination rate sharply declined after 6 months. The observation that AF begets AF is not new, nor is the observation that with prolonged duration of AF, the substrate becomes more complex [23, 24]. Therefore, together with a good method for mapping and targeting drivers of persistent AF, early timing may also be of the essence.

### Study limitations

This study has several important limitations. Data collection was limited to variables that were routinely collected and was incomplete in some patients. The studied population was small, and no comparisons with other ablation strategies or mapping systems were made. Therefore, conclusions with regard to safety and efficacy cannot be drawn based on this study alone, and our report should be interpreted as a feasibility pilot study.

## Conclusion

The AcQMap System is able to provide fast, high-resolution activation maps of persistent AF and atypical atrial flutter. Despite a high acute success rate, the recurrence rate of persistent AF in this selected, therapy-resistant population was relatively high. This may be due to the limited experience in the optimal use of this novel system and the selection of a very difficult patient population that had failed all prior treatment strategies.

## Supplementary Information

**Tab. S1** Procedural data of 14 patients treated for persistent atrial fibrillation and 7 patients treated for atypical atrial flutter (procedural time, radiofrequent ablation time, initial rythm, cycle length, ablation sites, acute succes, complications)

**Fig. S1** Number of activation patterns of interest by location in 14 patients with persistent atrial fibrillation

**Video** Example of right-sided atypical flutter in patient 16
