# Supplementary material for: Initial experience with AcQMap catheter for treatment of persistent atrial fibrillation and atypical atrial flutter
Source: Neth Heart J. 2021 Oct 26;30(5):273–81. doi: 10.1007/s12471-021-01636-w (PMC9043165; doi:10.1007/s12471-021-01636-w)
Supplement: Supplementary file 1 — Tab. S1 Procedural data of 14 patients treated for persistent atrial fibrillation and 7 patients treated for atypical atrial flutter (procedural time, radiofrequent ablation time, initial rythm, cycle length, ablation sites, acute succes, complications) [file 12471_2021_1636_MOESM1_ESM.docx]

**Tab. S1** Procedural data of 14 patients treated for persistent atrial fibrillation and 7 patients treated for atypical atrial flutter

| **Patient** | **PT, min** | **RFT, min** | **Initial rhythm** | **CL, ms** | **Ablation sites** | **Acute success** | **Complications** |
| --- | --- | --- | --- | --- | --- | --- | --- |
| 1 | 150 | 23 | AF | - | Posterior, anteroseptal | Yes | No |
| 2 | 180 | 16 | AF | - | LIPV, RIPV, RSPV, RIPV=>LIPV | Yes | No |
| 3 | 205 | 9 | AFL | 200 | IAS=>VCS | Yes | No |
| 4 | 220 | NA | AF | - | 4x PV, roof line, trigonum line | Yes | No |
| 5 | 42 | 7 | AF | - | LSPV, RSPV | Yes | No |
| 6 | 330 | 180 | AF | - | 4x PV, anteroseptal, MVA=>RIPV, anterior roof=>LSPV | No | Air embolus with transient ST segment elevations |
| 7 | 180 | 120 | AF | - | 4x PV, 2x posterior, anterior | Yes | No |
| 8 | 245 | 65 | AF | - | 4x PV, 2x septal, posterior=>RIPV, posterior=>LIPV, anterior | No | No |
| 9 | NA | NA | AFL | NA | RSPV=>mid roof, MVA=> TP, anterior | Yes | No |
| 10 | 200 | 53 | AF | - | Posterior, anterior IAS, low IAS, SC=>LIPV, LIPV=>RIPV, roof=>MVA, LSPV=>RSPV, septal roof line | Yes | No |
| 11 | 145 | NA | AF | - | RIPV, anterior=>MVA, posterior=>RIPV, RSPV=>MVA | Yes | No |
| 12 | 153 | NA | AF | - | Anterior roof, low posterior | Yes | No |
| 13 | 180 | 12 | AFL | 285 | RIPV=>MVA | Yes | No |
| 14 | 220 | 27 | AFL | 251 | Posterior | Yes | No |
| 15 | 180 | 35 | AF | - | low septum, septum=>MVA, mid septal RA | No | Inguinal bleeding |
| 16 | 240 | 10 | AFL | 200 | Septum, septum RA=>TVA | No | IAT for hemi paralysis, pacemaker implantation for complete atrioventricular block |
| 17 | 210 | NA | AF | - | RIPV, anterior, low posterior | Yes | No |
| 18 | 165 | NA | AF | - | LSPV, posterior, septum, roof=>LSPV | Yes | No |
| 19 | 200 | NA | AFL | NA | RIPV, RIPV=>LIPV, LIPV=>MVA | Yes | No |
| 20 | 180 | NA | SR | 260 | Septum=>MVA, posterior septum=>TVA, high septum=>TVA, lateral RA | Yes | Pacemaker implantation for complete atrioventricular block |
| 21 | 240 | NA | AF | - | RIPV=>MVA, VCS=>posterior, anterior=>VCS=>TVA | Yes | Inguinal bleeding |
| AFL = atrial flutter, CL = cycle length, IAS = intra atrial septum, IAT = intra –arterial thrombolysis, LIPV = left inferior PV, LSPV = left superior PV, MVA = mitral valve annulus, PT = procedural time, PV = pulmonary vein, RA = right atrium, RFT = radiofrequent ablation time, RIPV = right inferior PV, RSPV = right superior PV, SR = sinus rhythm, TVA = tricuspid valve annulus, VCS = vena cava superior. Ablation sites are left sided unless otherwise stated. => indicates an ablation line in between. | | | | | | | |
