# Supplementary figures and images for: Initial experience with AcQMap catheter for treatment of persistent atrial fibrillation and atypical atrial flutter
Source: Neth Heart J. 2021 Oct 26;30(5):273–81. doi: 10.1007/s12471-021-01636-w (PMC9043165; doi:10.1007/s12471-021-01636-w)

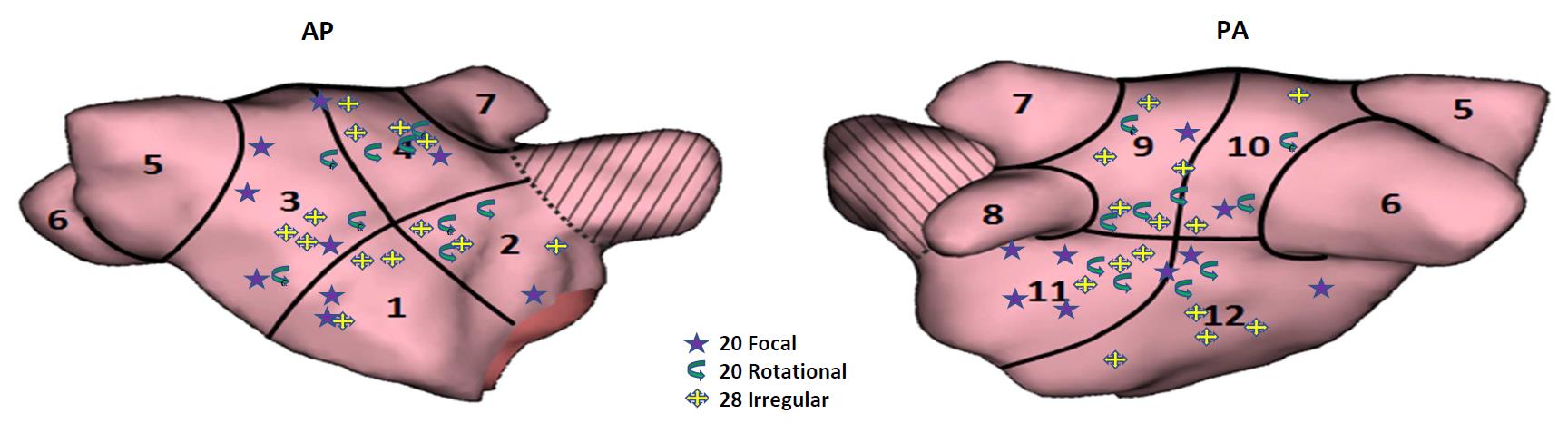

Supplement: Supplementary file 2 — Fig. S1 Number of activation patterns of interest by location in 14 patients with persistent atrial fibrillation [file 12471_2021_1636_MOESM2_ESM.jpg]
